# A brief overview of BNIP3L/NIX receptor‐mediated mitophagy

**DOI:** 10.1002/2211-5463.13307

**Published:** 2021-10-11

**Authors:** Mija Marinković, Ivana Novak

**Affiliations:** ^1^ School of Medicine University of Split Croatia

**Keywords:** BNIP3L/NIX, mitochondria, mitophagy, reticulocytes

## Abstract

Mitophagy is a form of autophagy specialized to selectively remove mitochondria. Although the PINK1/Parkin pathway is the best described mitophagy of damaged mitochondria, receptor/mediated mitophagy seems to have a pivotal role in cellular development and specialization. The most studied mitophagy receptor BCL2/adenovirus E1B 19‐kDa‐interacting protein 3‐like (BNIP3L/NIX) is shown to be important for the programmed removal of healthy mitochondria during terminal differentiation of erythrocytes, but its role has been proven in various cell types. Despite recent advances in our understanding of its regulation by phosphorylation and dimerization, there remain numerous questions on how BNIP3L/NIX tightly balances between cellular life and death decisions. This brief review intends to summarize ongoing dilemmas related to BNIP3L/NIX.

## Graphical abstract

The inactive form of the BCL2/adenovirus E1B 19‐kDa‐interacting protein 3‐like (BNIP3L/NIX) receptor is found as a monomer phosphorylated on C‐terminal serine 212. Upon mitophagy induction, the receptor is dephosphorylated and this allows BNIP3L/NIX monomers to form the active form of the receptor, BNIP3L/NIX dimer, which is also additionally activated with double phosphorylation near the LIR domain. Dimerization enables stronger recruitment of autophagosomes on selected mitochondria and more efficient mitophagy.

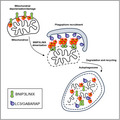

**Abbreviations**
AMBRA1: activating molecule in BECN1‐regulated autophagy protein 1
Atg: autophagy‐related genes
BCL2: B‐cell lymphoma 2
BCL2L13: Bcl2‐like 13
BH3: Bcl‐2 homology 3
BNIP3: BCL2/adenovirus E1B 19‐kDa‐interacting protein 3
BNIP3L/NIX: BCL2/adenovirus E1B 19‐kDa‐interacting protein 3‐like
CK2: casein kinase 2
FKBP8: FK506 binding protein 8
FUN14: domain‐containing protein 1
FUNDC1: FUN14 domain‐containing protein 1
GABARAP: GABA type A receptor‐associated protein
LC3: light chain 3
LIR: LC3‐interacting region
MIRO: mitochondrial Rho GTPase
NBR1: neighbor of BRCA1 gene 1
NDP52: nuclear domain 10 protein 52
OMM: outer mitochondrial membrane
OPTN: optineurin
PGAM5: phosphoglycerate mutase 5
PHB2: Prohibitin 2
PINK1: PTEN‐induced putative kinase 1
RGC: retinal ganglion cells
SQSTM1: sequestosome‐1
SRC: steroid receptor coactivator
TAX1BP1: TAX1 binding protein 1
TBK1: Tank‐binding kinase 1
VDAC: voltage‐dependent anion channel 1

Integrity maintenance is one of the primary goals of a cell. The balance between the production and destruction of cellular molecules and organelles presents one of the most demanding assignments. It is well known that the process of autophagy is the most effective in the removal of large components that are either not needed or are damaged or dysfunctional. The complex mechanisms of bulk and selective autophagy, from initiation, progression, and finally degradation, require tight and precise regulation. Furthermore, dysregulation of autophagy easily leads to cellular malfunction that can manifest as cancer development, tissue degeneration, premature aging, or many other pathologies. Therefore, it is not surprising that autophagy is one of the most studied cellular processes. Here, we discuss one of the mechanisms of receptor‐mediated mitophagy: selective removal of mitochondria.

## Mitophagy as a mitochondrial quality control mechanism

**Fig. 1:**
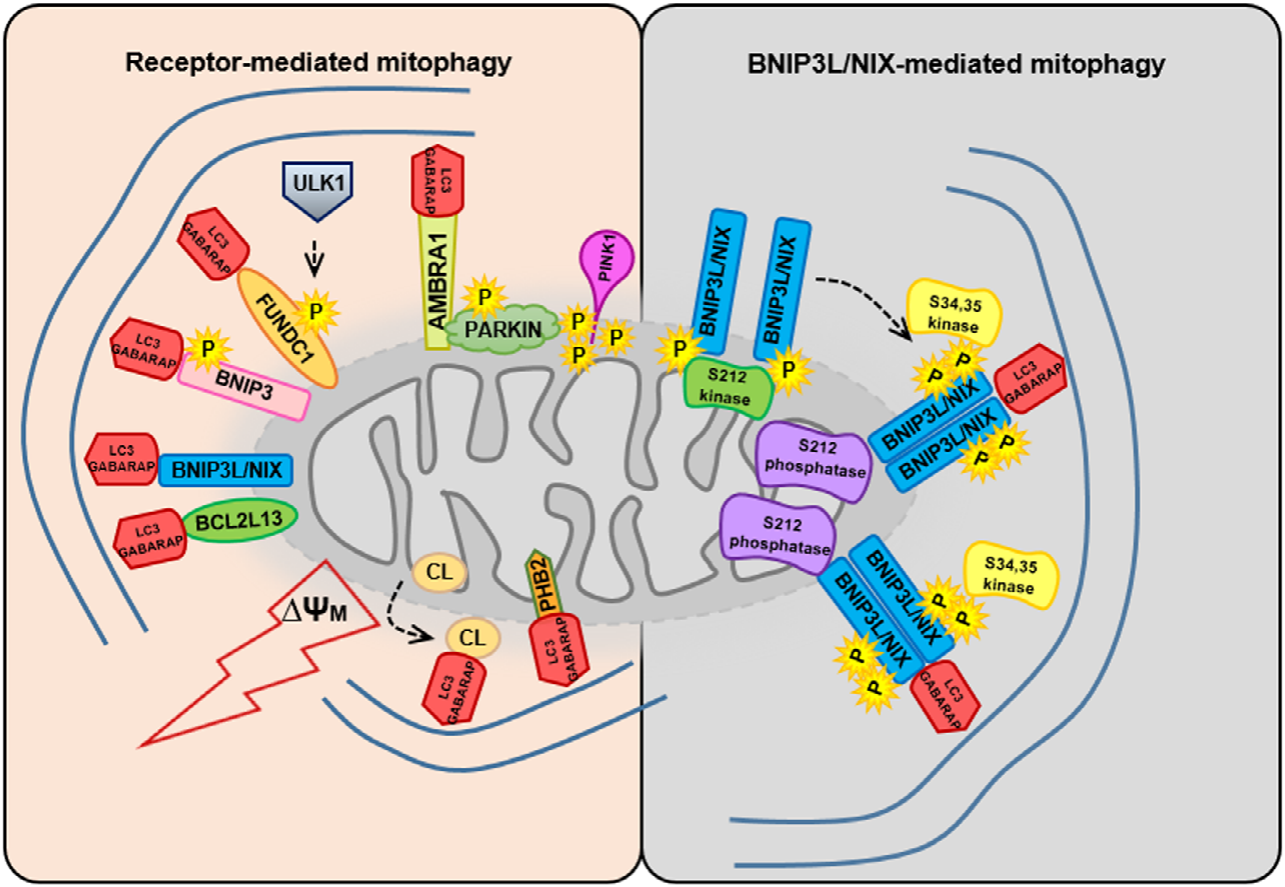
Molecular mechanisms of receptor‐mediated mitophagy. Receptor‐mediated mitophagy mechanisms are regulated by interactions between outer membrane (BNIP3, BNIP3L/NIX, FUNDC1, BCL2L13, AMBRA1) or inner membrane (PHB2) mitochondrial receptors, which directly bind LC3/GABARAP proteins located on the autophagosomal membrane through the conserved LC3‐interacting region. Different phosphorylation events strictly regulate mitophagy initiation and progression (left). The BNIP3L/NIX‐mediated mitophagy mechanism is described in detail on the right part of the figure. Upon mitophagy induction, Ser212‐phosphorylated BNIP3L/NIX monomers are dephosphorylated at the C terminus to form more stable BNIP3L/NIX dimers. In parallel, double LIR phosphorylation (Ser34, and Ser35) enhances autophagosomal recruitment on mitochondria.

A vast mitochondrial network acts as the powerhouse of every cell. Even though mitochondria are best known for this function, through which they serve as the major supply of adenosine triphosphate, their structural complexity hides several different roles essential for a cell, from regulation of cellular calcium levels, proliferation and apoptosis, steroid, and heme synthesis to cellular quality control. Due to these demanding tasks, it is crucial to maintain a healthy and functional mitochondrial population. Dynamic mitochondrial fusion and fission are capable of keeping the mitochondrial network functional to a limited extend [1]; however, when the damage is too large or massive removal of mitochondria is needed, mitophagy is the process that takes charge. Activation of mitophagy can be triggered either by different stress conditions or as programmed mitochondrial removal. In the case of induction by stress, the PTEN‐induced putative kinase 1 (PINK1)/Parkin response pathway is activated [2], while during programmed degradation of mitochondria, the major players are mitophagy receptors (Fig. 1) [3]. The PINK1/Parkin pathway (where PINK1 is a kinase and Parkin an E3 ligase) is specialized in the removal of damaged mitochondria through a series of events that start with the stabilization of PINK1 at the outer mitochondrial membrane (OMM), caused by the loss of mitochondrial transmembrane potential; PINK1 then phosphorylates ubiquitin, which in turn recruits Parkin to a mitochondrial membrane [4]. This leads to PINK1‐dependent phosphorylation of Parkin that becomes activated to further ubiquitinate OMM proteins, including voltage‐dependent anion‐selective channel (VDAC) and mitochondrial Rho GTPase (MIRO) proteins [5, 6, 7]. Chen *et al*. have previously summarized the current knowledge of this pathway [8].

PINK1/Parkin‐independent mitophagy pathways involve receptors primarily localized at the OMM, but inner membrane receptors have also been described [9]. All receptors share the common feature of recruiting autophagic machinery to mitochondria by binding to light chain 3 (LC3)/GABA type A receptor‐associated protein (GABARAP) proteins through a conservative LC3‐interacting region (LIR) domain, and abrogation of this interaction enables the receptors to activate mitophagy.

## BNIP3L/NIX—a pioneer of receptor‐mediated mitophagy

Although mitophagy primarily acts as a mitochondrial quality control mechanism required for the removal of damaged and dysfunctional mitochondria, its role in the development of specialized cell types remains insufficiently understood, but certainly worthy of further research. So far, the best described example of the programmed developmental function of mitophagy is complete removal of mitochondria during terminal mammalian erythropoiesis, when reticulocytes undergo intense intracellular remodeling to become mature erythrocytes [10, 11, 12, 13, 14]. Interestingly, these mitochondria are completely functional but not necessary for particular cell types, such as erythrocytes. For that reason, the molecular mechanism of programmed mitophagy activation during development and differentiation differs from quality control mitophagy and depends on selective autophagy receptors. Undoubtedly, the first BCL2/adenovirus E1B 19‐kDa‐interacting protein 3‐like (BNIP3L/NIX) [B‐cell lymphoma 2 (BCL2)/adenovirus E1B 19‐kDa‐interacting protein 3‐like] mitophagy receptor to be described [15] has a central role in terminal mammalian erythropoiesis. It was shown that Nix^−/−^ mice exhibit ineffective mitochondrial removal that inhibits normal erythrocyte maturation and causes anemia [16, 17]. Erythrocytes are not the only cell type that eliminates mitochondria during differentiation. Programmed BNIP3L/NIX‐dependent mitochondrial elimination regulates neurogenesis of retinal ganglion cells (RGCs) and proinflammatory macrophages [18]. Furthermore, BNIP3L/NIX‐mediated mitophagy plays a critical part in the differentiation process of cardiac progenitor cells to form a mature mitochondrial network inside cardiomyocytes. During differentiation of cardiac progenitor cells, both BNIP3L/NIX and FUNDC1 are upregulated and indispensable for the formation of a functional interconnected mitochondrial network. Knockdown of mitophagy receptors BNIP3L/NIX and FUNDC1 in these cells leads to mitochondrial fission and accumulation of dysfunctional donut‐shaped mitochondria with a greater susceptibility to oxidative stress‐mediated cell death [19]. Since mitochondria are the most abundant organelles in cardiomyocytes and are imperative for the maintenance of function, proper mitophagy is crucial for the regulation of cardiomyocyte activity. Furthermore, a recent study revealed that dysregulation of mitophagy in hippocampal neurons is initiated by stress‐induced levels of glucocorticoids and subsequent BNIP3L/NIX reduction. BNIP3L/NIX downregulation induces synaptic dysfunction arising from the accumulation of damaged mitochondria that leads to reduced mitochondrial respiration function and synaptic density [20]. It is also worth emphasizing that BNIP3L/NIX‐mediated mitophagy is a fundamental mechanism by which intestinal epithelial cells diminish mitochondrial stress and dysfunction during intestinal inflammation [21]. Additionally, it was found that BNIP3L/NIX‐driven mitochondrial breakdown signaling is increased in skeletal muscles of chronic obstructive pulmonary disease patients, and this is also associated with disease severity [22]. Finally, BNIP3L/NIX‐mediated mitophagy activity is also enhanced in disuse‐induced muscle atrophy as the main source of reactive oxygen species production responsible for muscle degeneration [23].

The elucidation of the mechanisms of receptor‐mediated mitophagy began with the discovery of yeast protein autophagy‐related genes (Atg)32, the only described mitophagy receptor in yeast at present [24, 25]. In contrast to yeast, multicellular organisms not only developed a system with several receptors for selective elimination of mitochondria, but also two different molecular mechanisms by which these receptors act, thus enabling precise regulation of the process: [26] via ubiquitin‐binding receptors and [27] via ubiquitin nonbinding receptors. Unlike adaptor proteins, p62/SQSTM1, optineurin (OPTN), nuclear domain 10 protein 52 (NDP52), TAX1 binding protein 1 (TAX1BP1), and neighbor of BRCA1 gene 1 (NBR1) have to bind K63 poly‐ubiquitin chains on mitochondria before binding LC3/GABARAP proteins; the action of mitophagy receptors does not depend on direct ubiquitination. Moreover, mitophagy receptors of this type are located on the OMM and contain the LIR motif on the cytoplasmic side for direct recruitment of growing autophagosomes on mitochondria without the need for adaptors (Fig. 1). The aforementioned Atg32‐dependent mitophagy in yeast is quite similar to such receptor‐mediated mitophagy in mammals [28]. A real Atg32 homolog in mammals has not yet been described; however, several OMM proteins show identical functional characteristics to yeast Atg32: FUN14 domain‐containing protein 1 (FUNDC1), BCL2/adenovirus E1B 19‐kDa‐interacting protein 3 (BNIP3), BNIP3L/NIX, Bcl2‐like 13 (BCL2L13), FK506 binding protein 8 (FKBP8), and activating molecule in BECN1‐regulated autophagy protein 1 (AMBRA1) [15, 16, 17, 27, 29, 30]. In addition, the mitophagy receptors Prohibitin 2 (PHB2) [31] and cardiolipin [26], identified on the inner mitochondrial membrane, can also directly bind LC3/GABARAP proteins and recruit the growing autophagosome to mitochondria (Fig. 1). Here, we discuss the best studied mitophagy receptor: BNIP3L/NIX.

Initial studies of BNIP3L/NIX protein have focused on its proapoptotic function, since BNIP3L/NIX was generally classified as a protein able to stimulate apoptosis (programmed cell death) due to its single Bcl‐2 homology 3 (BH3) domain [32]. The last decade has been a period of intensive BNIP3L/NIX research, but primarily in the context of mitophagy. Indeed, from the first findings of the importance of the BNIP3L/NIX receptor in programmed mitochondrial elimination during terminal reticulocyte differentiation, we have since learned a substantial amount about the molecular basis of mitochondrial selectivity. Despite this, the dual function of the BNIP3L/NIX receptor, concerning cellular life (mitophagy) versus death (apoptosis) fate, is still unclear, particularly its molecular regulation. Mitochondrial removal in reticulocytes depends on the recruitment of autophagosomes via the LIR domain at the N‐terminal end of the BNIP3L/NIX protein. However, a detailed understanding of the molecular mechanisms underlying the upstream signals that lead to BNIP3L/NIX activation or the downstream mechanism of action of the activated receptor are lacking. In addition, it is unclear how damaged mitochondria are selected and separated from healthy ones to ultimately be degraded by selective autophagy. Moreover, the big question remains: is there any difference between the molecular mechanisms that underly (a) the removal of damaged mitochondria and (b) the removal of functional mitochondria during erythrocyte differentiation?

Novak *et al*. [15] described BNIP3L/NIX as a selective autophagy receptor that binds to Atg8 homologs, LC3/GABARAP proteins, through a conserved LC3‐interacting region (LIR) motif at the amino terminus of BNIP3L/NIX [33, 34]. Briefly, they have shown how disruption of LIR disables the interaction between BNIP3L/NIX and LC3/GABARAP that consequently leads to impaired mitophagy. Mitophagy is not completely lost and a portion of mitochondria still can be removed; however, the differentiation of erythrocytes is disturbed. A few years later, phosphorylation‐driven regulation of the BNIP3L/NIX:LC3B interaction was discovered to be due to phosphorylation of two serines, Ser34 and Ser35, juxtaposed to the BNIP3L/NIX LIR, which in turn enhances mitophagy receptor engagement (Fig. 1) [35]. LIR phosphorylation, as a possible mechanism of selective regulation of autophagy receptor activity, was first described by Wild *et al*. (2011) for another autophagy and mitophagy receptor OPTN. Phosphorylation of five adjacent serines upstream of the OPTN LIR domain contributes to stronger receptor activation and more efficient xenophagy of ubiquitinated cytosolic Salmonella [36]. Subsequent studies also confirmed analogous mechanisms for BNIP3, p62, NDP52, and TAXBP1 receptors. Except for BNIP3 and BNIP3L/NIX, LIR phosphorylation occurs through the action of Tank‐binding kinase 1 (TBK1) [36, 37, 38]. Since mitochondria, according to endosymbiotic theory, originated from aerobic bacteria, some parts of the xenophagy and mitophagy mechanisms may be evolutionarily conserved between bacteria and mitochondria.

Today, we know the biophysical nature of interactions formed between the phosphorylated BNIP3L/NIX receptor and autophagosomal proteins LC3/GABARAP [35]. Interestingly, those interactions are almost identical to the observed interactions established between the p62 receptor and LC3, as well as between Atg19 and Atg8, the yeast homolog of previously mentioned p62 and LC3 [39]. This again indicates possible evolutionary conservation of molecular mechanisms of various forms of selective autophagy.

Seemingly, LIR phosphorylation is a universal mechanism of activation for most selective autophagy receptors. Xenophagy receptors OPTN and NDP52 are phosphorylated by the action of TBK1 kinase l, and this phosphorylation enhances Salmonella removal from infected cells [36, 37]. Furthermore, phosphorylation of the Ser17 and Ser24 of BNIP3 contributes to more efficient LC3B and GABARAP‐L2 binding and induces mitophagy, but also defines the fate of BNIP3 protein as a mitophagy receptor. In fact, in the absence of LIR phosphorylation, the BNIP3 receptor acts as a usual proapoptotic BH3 protein [38]. Still, the only known exception is the FUNDC1 mitophagy receptor, whose phosphorylation has a negative effect on LC3/GABARAP binding and mitophagy activation [29]. Under physiological conditions, Ser13 on FUNDC1 is phosphorylated by the action of Casein kinase 2 (CK2), while Tyr18 is phosphorylated by steroid receptor coactivator (SRC) kinase (Src‐family kinase). These phosphorylation events most likely inhibit the usually stable interaction between receptor and LC3 proteins and prevent mitophagy. During hypoxia, phosphatase phosphoglycerate mutase 5 (PGAM5) dephosphorylates the receptor, which is then capable of binding to LC3 proteins and activating mitophagy [29, 40]. This opposite effect of LIR phosphorylation on mitophagy activation between FUNDC1 and all other mitophagy receptors arises from the structural characteristics of the complex receptor LC3B, and indicates the possibility of specific regulation of individual forms of receptor‐induced mitophagy depending on spatial and temporal differences in the presence of certain types of mitophagy receptors and autophagy proteins.

Since functional LIR domain is not enough to fully activate mitophagy during terminal erythropoiesis in mammals [15], additional mechanisms for BNIP3L/NIX‐mediated mitochondrial removal must exist and are probably not LIR‐dependent. In polyacrylamide gels, BNIP3L/NIX protein, similar to its homolog BNIP3, migrates as a monomer of ˜ 42 kDa and as a stable and dominant dimer form with a size of ˜ 80 kDa. The functional importance of these BNIP3L/NIX dimers has been recently described [41]. Allegedly, stable BNIP3L/NIX homodimers bind autophagosomal protein LC3 more strongly, and also recruit autophagosomes on mitochondria more robustly than its monomeric form. Furthermore, BNIP3L/NIX dimerization is not only important for mitophagy initiation, but also has an essential role in the final phase during mitochondrial removal. Marinković *et al*. have further shown that the BNIP3L/NIX transmembrane domain is responsible for receptor dimerization, since disruption of dimerization, by mutations in the transmembrane domain, causes a similar effect on mitophagy as LIR disruption. Additionally, Marinković *et al*. [41] identified that dephosphorylation of Ser212 in the intramembrane C‐terminal part of the receptor is a key regulatory upstream signal that controls dimerization (Fig. 1). This novel mechanism of receptor dimerization is comparable to the previously described tendency of the p62 selective autophagy receptor to oligomerize during the removal of damaged mitochondria [42, 43] or protein aggregates in mammals [44]. The possibility that BNIP3L/NIX also creates oligomers is not excluded, especially if we acknowledge that BNIP3L/NIX was initially described as one of the pro‐apoptotic mitochondrial membrane proteins that often has an affinity to form oligomeric structures. Interestingly, Wu *et al*. described how BNIP3L/NIX was degraded by the proteasome in ischemic neurons and brains, which induced mitophagy deficiency, thereby demonstrating the importance of Ser212 phosphorylation. However, this deficiency could be rescued and cerebral ischemic injury prevented if dimerized BNIP3L/NIX was added or proteasomal degradation of BNIP3L/NIX was blocked [45]. This further proved the importance of dimerization as a regulation mechanism of receptor‐mediated mitophagy in different cell types, and suggests BNIP3L/NIX dimerization and mitophagy reactivation as an excellent therapeutic strategy for neuronal recovery after cerebral ischemic injury.

## Perspectives

The knowledge gained in the last decade in the field of mitophagy has brought us closer to understanding how damaged or unnecessary mitochondria are removed. More than one mechanism of mitophagy has been revealed, and here, we have summarized reports that show BNIP3L/NIX receptor‐mediated mitophagy is indispensable for the differentiation of reticulocytes, also called development‐induced mitophagy. The discovery that regulation of BNIP3L/NIX occurs through phosphorylation of LIR at its cytoplasmic side, together with the phosphorylation of the intermembrane part that defines its dimerization status, has revealed new challenges in the field. From this cognition, many questions have risen: Could we identify kinases and phosphatases that are involved at these two phosphorylation diverse sites? What are the upstream signals that activate phosphorylation events? How is BNIP3L/NIX proapoptotic function blocked during its role in mitophagy? What is the impact on reticulocyte development when BNIP3L/NIX mitophagy is not functional? Do myeloproliferative disorders (such as polycythemia vera, erythrocytosis, or myelofibrosis) develop due to abnormal mitophagy? What is the role of receptor‐mediated mitophagy in cardiomyopathies or cerebral ischemic injury? Is nonfunctional mitophagy druggable? Even though our knowledge is already excellent, a vast amount of information still needs to be unveiled to fully understand cellular behavior and human pathologies related to mitophagy in general, as well as receptor‐mediated mitophagy.

## Conflict of interest

The authors declare no conflict of interest.

## Author contributions

MM and IN conceived the concept and wrote the paper.
